# Supplementary material for: EP300 deficiency leads to chronic replication stress mediated by defective replication fork protection
Source: Nat Commun. 2025 Dec 7;17:475. doi: 10.1038/s41467-025-67171-z (PMC12800293; doi:10.1038/s41467-025-67171-z)
Supplement: Supplementary file 1 — Supplementary Information [file 41467_2025_67171_MOESM1_ESM.pdf]

Supplementary Figures and Tables

Supplemental Figures

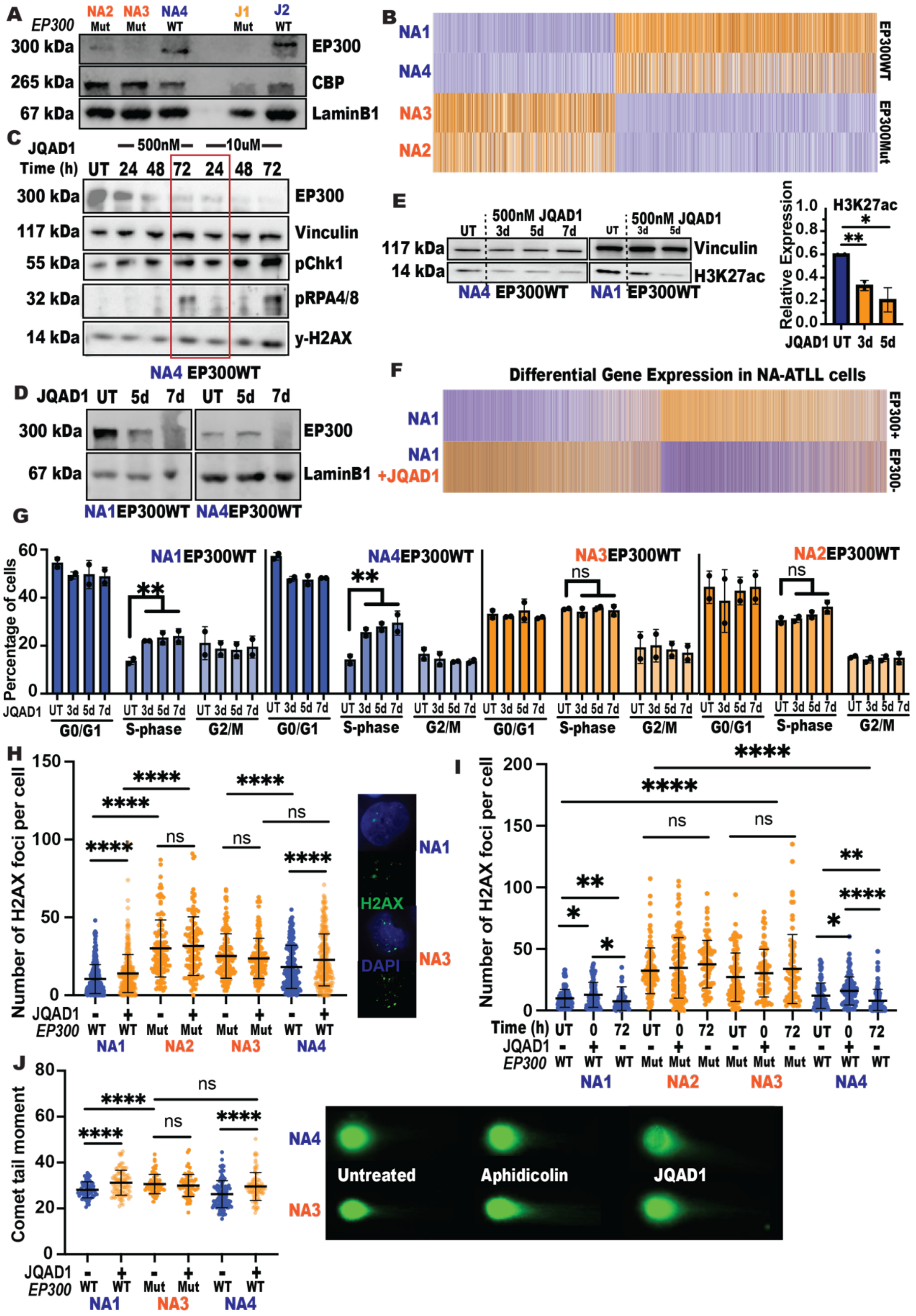

Supplemental Figure 1: EP300-mutated cells with intact CBP have decreased histone acetylation, altered transcriptional programs, and elevated DNA damage. (A) Immunoblot depicting EP300 and CBP total protein levels from WCE in EP300Mut and EP300WT NA-ATLL cells (B) Heatmap demonstrating gene expression

patterns of top 100 differential genes in EP300WT (NA1, NA4) compared to EP300Mut (NA2, NA3) cells; (C) Immunoblot demonstrating time-dependent (0, 24h, 48h, 72h) loss of EP300 after low-dose (500nM) and high-dose (10uM) JQAD1 treatment; (D) Immunoblot demonstrating time-dependent loss of EP300 protein- 3,5,7 days post 500nM JQAD1 treatment in EP300WT NA-ATLL cell lines (NA1, NA4); (E) Left: Expression levels of H3K27 histone acetylation mark by immunoblotting 3,5,7 days post 500nM JQAD1 treatment (48h) in two EP300WT NA-ATLL cells. Expression levels of Vinculin were used as a loading control. Right: A histogram showing the relative expression of H3K27ac. p-values * <0.03. (F) Heatmap demonstrating gene expression patterns of top 100 differential genes in EP300WT (NA1) +/- JQAD1; (G) Histogram summarizing the percentage of cells in different stages of the cell cycle (G0/G1, S,G2/M) 3, 5 and 7 days post 500nM JQAD1 treatment (48h), quantified from propidium Iodide (PI) based cell cycle analysis by flow cytometry in EP300WT cell lines (NA1, NA4) and EP300Mut cells (NA2, NA3); (H) Analysis of the number of pH2AX foci per cell nuclei in EP300WT and EP300Mut cells -/+ 500nM JQAD1 treatment (48h), N=2; (I) Analysis of the number of pH2AX foci per cell nuclei in EP300WT and EP300Mut -/+ 500nM JQAD1 treatment (48h) and 72h after release from 500nM JQAD1, N=2; (J) Measurement of DNA single-strand breaks by alkaline Comet assay in EP300Mut and EP300WT NA-ATLL cells treated with 500nM JQAD1 (48h). Comet tail lengths were measured using the OpenComet plugin as part of the ImageJ software, N=2. Representative images are on the right. For all experiments, data are presented as mean values +/- SD. P-values were determined by a two-tailed Student's T-test. The p-values are indicated as follows: * <0.03, ** <0.0021, *** <0.0002, **** <0.0001. Scale bar 10 µm. N represents two/three experimental replicates from independent cultures of cells. Source data are provided as a Source Data file.

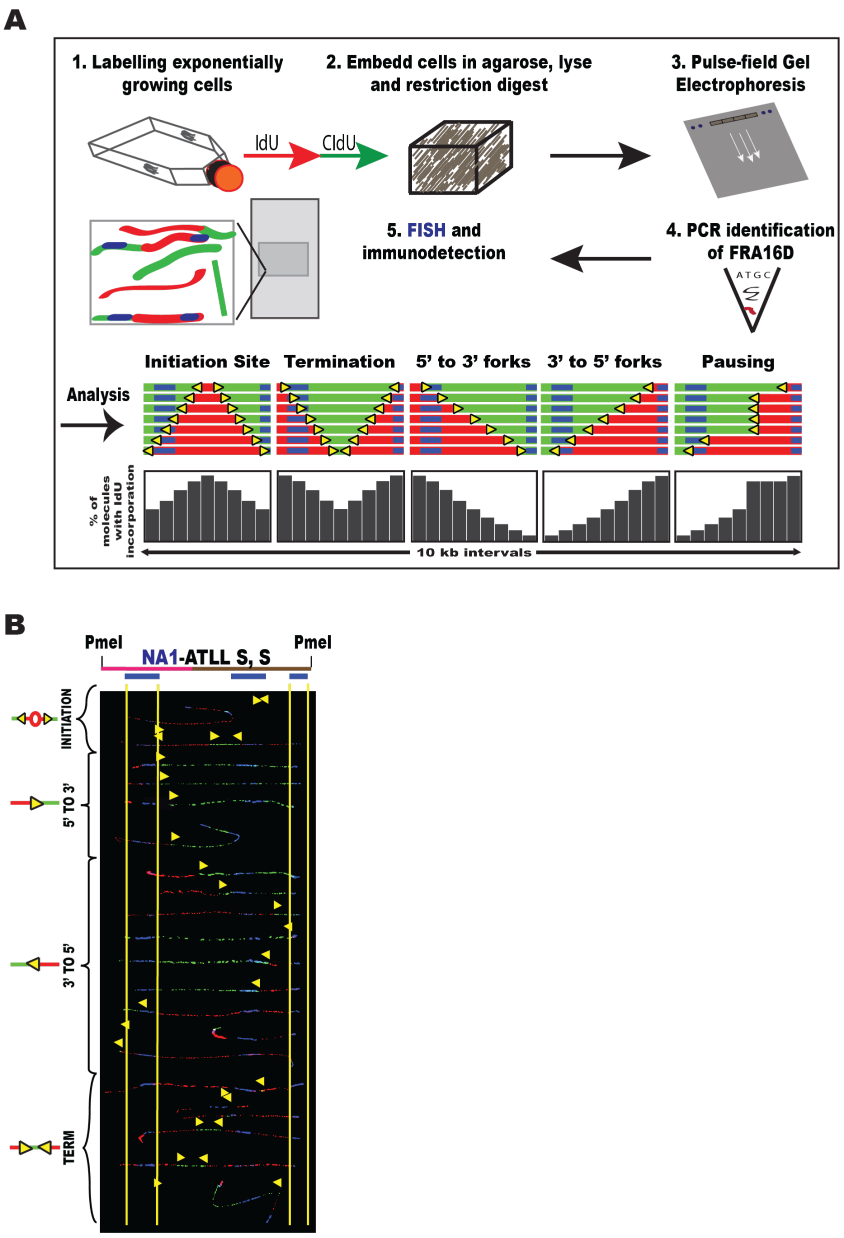

Supplemental Figure 2: Schematic of single-molecule analysis of replicated DNA (SMARD), related to Figure 2. (A) Schematic representation of the various stages of SMARD. Cells are pulsed with nucleoside analogs (IdU-green; CldU-red) and embedded in agarose plugs. The cells are first lysed; proteins are digested by proteinase K and then subjected to restriction digestion. The restriction-digested DNA is resolved by pulse field gel electrophoresis. The slice containing the FRA16D locus is identified by PCR analysis. The agarose from the identified slice is melted, and the DNA is stretched onto silanized glass slides. Biotinylated FISH probes are used for identification of the fragment, and immunostaining is utilized to visualize the IdU tract in red, the CldU tract in green, and the FISH probes in blue. The resulting molecules are arranged to yield recognizable replication patterns (from the left): initiating molecules, terminating molecules, replication forks travelling in the

3’ to 5’ and 5’ to 3’ direction, which are easily interpreted by the IdU incorporation histograms. Schematic was generated using PowerPoint. (B) Aligned photomicrograph images of labeled DNA molecules from the DNA replication program at CFS-FRA6E in EP300WT cells treated with negative control (S, S) stereoisomer. Source data are provided as a Source Data file.

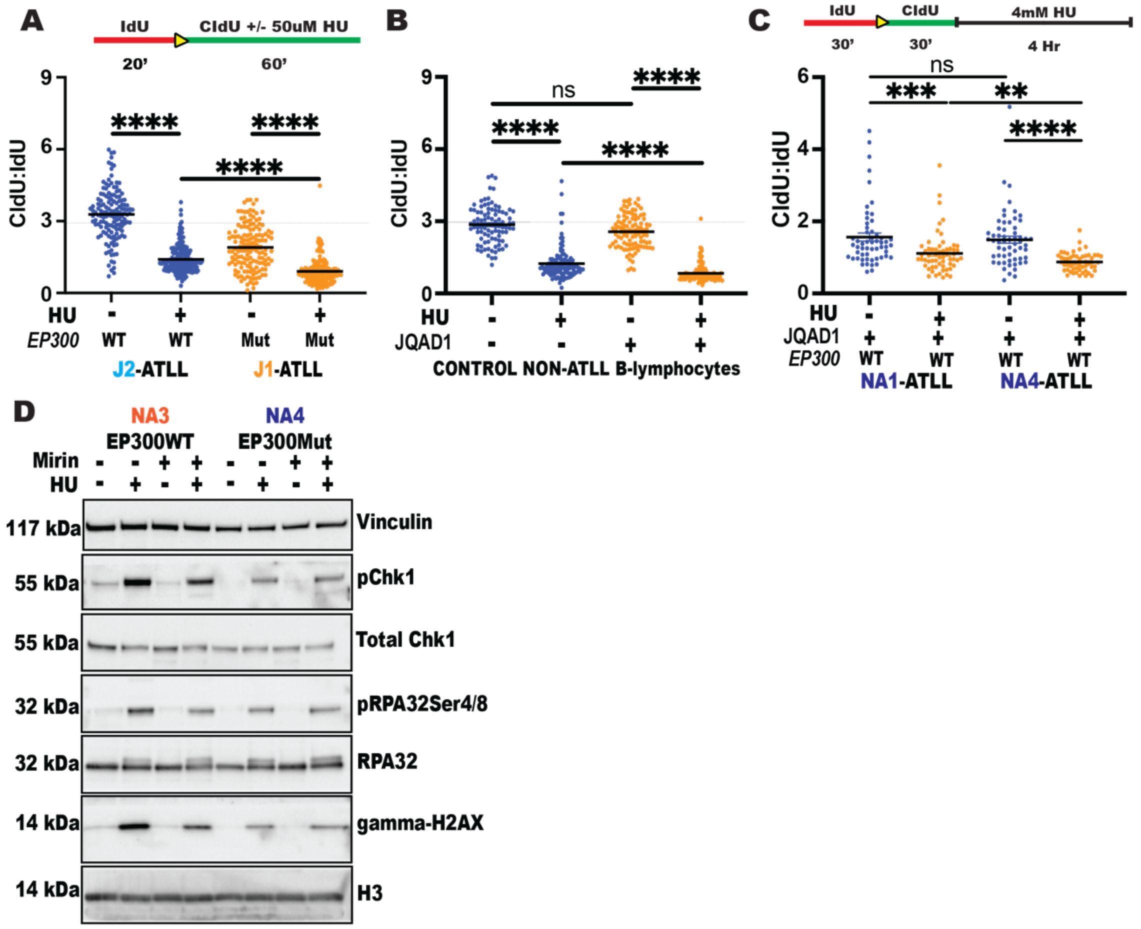

Supplemental Figure 3: EP300 deficiency results in genome-wide nucleolytic degradation. (A) DNA fiber analysis of 50uM hydroxyurea (HU) (60min) treated EP300WT (J2) and EP300Mut (J1) to assess replication fork stalling, N=3. (B) DNA fiber analysis measuring nucleolytic degradation after 4mM HU treatment (30min) in control non-ATLL EP300WT B-lymphocyte cells +/- 500nM JQAD1 (48h). EP300WT cells treated with the negative control (S, S) stereoisomer (500nM for 48h) were used as the control group for all JQAD1 experiments. The fork rate (CldU/IdU ratio) is indicated (N=3). For all experiments, data are presented as mean values +/- SD. P-values were determined by a two-tailed Student’s T-test. The p-values are indicated as follows: * <0.03, ** <0.0021, *** <0.0002, **** <0.0001. Scale bar 10 µm. (D) A. Expression levels of phospho-RPA Ser4/8 and phospho-Histone H2AX Ser139 in EP300WT (NA4) and EP300Mut NA-ATLL (NA3) cells treated with 2mM HU (4h), in the presence or absence of 50uM Mirin (4h), by western blotting. Expression levels of Vinculin were used as a loading control.

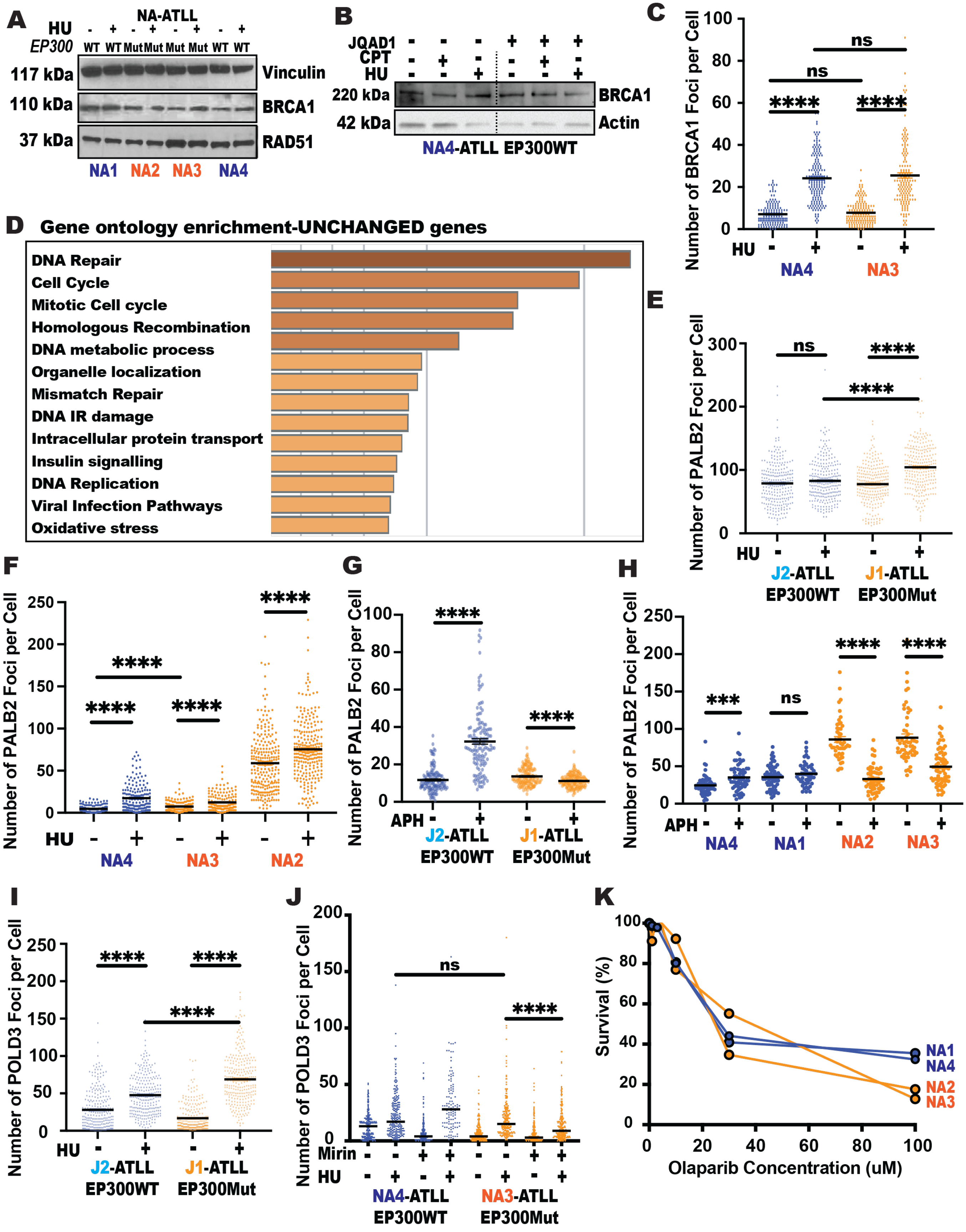

A
NA-ATLL
HU
EP300
WT WT Mut Mut Mut Mut WT WT
117 kDa
Vinculin
110 kDa
BRCA1
37 kDa
RAD51
NA1 NA2 NA3 NA4
B
JQAD1
CPT
HU
220 kDa
BRCA1
42 kDa
Actin
NA4-ATLL EP300WT
C
Number of BRCA1 Foci per Cell
ns
****
NA4 NA3
D
Gene ontology enrichment-UNCHANGED genes
DNA Repair
Cell Cycle
Mitotic Cell cycle
Homologous Recombination
DNA metabolic process
Organelle localization
Mismatch Repair
DNA IR damage
Intracellular protein transport
Insulin signalling
DNA Replication
Viral Infection Pathways
Oxidative stress
E
Number of PALB2 Foci per Cell
J2-ATLL EP300WT
J1-ATLL EP300Mut
F
Number of PALB2 Foci per Cell
NA4 NA3 NA2
G
Number of PALB2 Foci per Cell
APH
J2-ATLL EP300WT
J1-ATLL EP300Mut
H
Number of PALB2 Foci per Cell
***
NA4 NA1 NA2 NA3
I
Number of POLD3 Foci per Cell
J2-ATLL EP300WT
J1-ATLL EP300Mut
J
Number of POLD3 Foci per Cell
Mirin
NA4-ATLL EP300WT
NA3-ATLL EP300Mut
K
Survival (%)
Olaparib Concentration (uM)
NA1
NA4
NA2
NA3

Supplemental Figure 4: EP300 deficient cells have a prominent defect in downstream fork restart machinery. (A) Expression levels of BRCA1 and RAD51 proteins from WCE in NA-ATLL cells EP300WT/Mut cells treated with 2mM HU (4h) by immunoblotting. Expression levels of Vinculin were used as a loading control; (B) Expression levels of BRCA1 protein from WCE in EP300WT NA4 cells treated with 2mM HU (4h) or 1uM Camptothecin (4h), in the presence or absence of 500nM JQAD1 (48h), by immunoblotting. Expression levels of Actin were used as a loading control. (C) Analysis of the number of BRCA1 foci per cell nucleus (DAPI, blue) in EP300WT and EP300Mut NA-ATLL cells exposed to 2mM HU (4h), N=3; (D) Gene ontology enrichment analysis demonstrating relatively unchanged genes in EP300WT and EP300Mut cells. (E-H) Analysis of the number of PALB2 foci per cell nucleus in EP300WT and EP300Mut J-ATLL cells (E,G) or NA-ATLL cells (F,H) exposed to either 2mM HU (4h) or 0.4uM APH (overnight), N=3; (I-J) Analysis of number of POLD3 foci per cell nucleus in EP300WT and EP300Mut J-ATLL cells exposed to 2mM HU (4h) (I) and NA-ATLL cells exposed to 2mM HU (4h), in the presence or absence of 50uM Mirin (4h) (J), N=3; For all experiments, data are presented as mean values +/- SD. P-values were determined by a two-tailed Student's T-test. The p-values are indicated as follows: * <0.03, ** <0.0021, *** <0.0002, **** <0.0001. Scale bar 10 μm. (K) Percentage survival calculated from invitro toxicity assays in response to varying concentrations of Olaparib (0.01, 1, 3, 10, 30 and 100uM) in EP300Mut/WT NA-ATLL cell lines over the course of 72 hours. Source data are provided as a Source Data file.

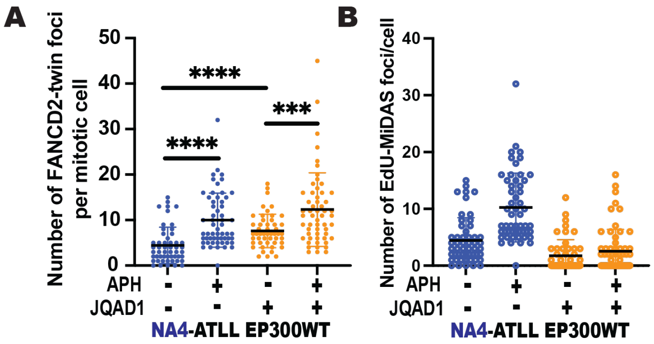

Supplemental Figure 5: EP300 deficient cells have under-replicated DNA and ineffective MiDAS. Analysis of FANCD2 twin-foci (A) and EdU foci (B) per mitotic cell in EP300WT exposed to negative control (S, S) stereoisomer or 500nM JQAD1 (4h), +/- 0.4uM APH (overnight), n=100 cells. For all experiments, data are presented as mean values +/- SD. P-values were determined by a two-tailed Student's T-test. The p-values are indicated as follows: * <0.03, ** <0.0021, *** <0.0002, **** <0.0001. Source data are provided as a Source Data file.

Supplemental Tables

Cell Line Name	Age at Diagnosis	Sex	EP300 mutations	CBP mutations	Reference PMID
NA1 (ATL13)	37	Female	None	None	30104217
NA2 (ATL18)	67	Female	Splice site SNP 4780-2 A>G	None	30104217
NA3 (ATL21)	37	Female	Splice site SNP 4286_6 C>T	None	30104217
NA4 (ATL29)	63	Female	None	None	30104217
J1 (ATL43Tb(-))	N/A	N/A	Non-synonymous SNP 4433 G>C, R1478P	N/A	30104217
J2 (ATL43T(+))	N/A	N/A	None	N/A	30104217

Supplemental Table 1. Mutational profile of ATLL cell lines

Gene Name	Log Fold Change	Log p-value
EXO1	1.55549535	9.03715732
FANCD2	0.99103375	4.88605665
ZRANB3	1.71181189	4.58838029
CHEK1	0.93522039	3.97421913
BRCA1	1.03575561	3.46713356
RIF1	0.71778435	3.35829294
RAD51C	0.81806419	2.35204588
EZH2	1.03375348	2.07576384
BRCA2	0.95592708	1.62774839
FANCM	0.73902722	1.47042576
MRE11A	0.67898429	1.3361923
RFWD3	0.69304955	1.22531996
PARP1	0.71184941	1.12094868
RAD51	0.55679044	0.93729654
ATRIP	0.47058956	0.84667709
FANCA	0.35636815	0.67640519
TIPIN	0.54485238	0.62976106
DNA2	0.7449019	0.61819308
RAD51D	0.5896556	0.6021993
TIMELESS	0.60884371	0.50777233
CHEK2	-0.7540697	0.50619977
RAD51B	0.52702658	0.4864578
DDX39A	0.41884996	0.38295053
TOP2A	0.39964542	0.34499906
TP53BP1	-0.3700749	0.21023491
VHL	0.29132829	0.18969018
SMARCAL1	-0.1213603	0.15491745
MUS81	0.08217825	0.07312984
HLTF	-0.193651	0.06649983
ATRX	0.07591362	0.0660276
POLD3	0.11951555	0.06341581
BOD1L1	0.04505223	0.03409017
ATR	-0.0384365	0.0248545
MRC1	-0.1720385	0.02001546

Supplemental Table 2. Transcriptomic changes to genes involved in fork protection. The highlighted genes are significantly altered in NA-ATLL EP300Mut cells (NA2 and NA3). Differential analysis was performed using DESeq2, and p-values were determined using the Wald test.
