# Supplementary material for: EP300 deficiency leads to chronic replication stress mediated by defective replication fork protection
Source: Nat Commun. 2025 Dec 7;17:475. doi: 10.1038/s41467-025-67171-z (PMC12800293; doi:10.1038/s41467-025-67171-z)
Supplement: Supplementary file 2 — Reporting Summary [file 41467_2025_67171_MOESM2_ESM.pdf]

nature portfolio

Corresponding author(s): Madireddy, Advaitha

Last updated by author(s): Oct 8, 2025

Reporting Summary

Nature Portfolio wishes to improve the reproducibility of the work that we publish. This form provides structure for consistency and transparency in reporting. For further information on Nature Portfolio policies, see our Editorial Policies and the Editorial Policy Checklist.

Statistics

For all statistical analyses, confirm that the following items are present in the figure legend, table legend, main text, or Methods section.

n/a	Confirmed	
☐	☒	The exact sample size (*n*) for each experimental group/condition, given as a discrete number and unit of measurement
☐	☒	A statement on whether measurements were taken from distinct samples or whether the same sample was measured repeatedly
☐	☒	The statistical test(s) used AND whether they are one- or two-sided<br>*Only common tests should be described solely by name; describe more complex techniques in the Methods section.*
☒	☐	A description of all covariates tested
☒	☐	A description of any assumptions or corrections, such as tests of normality and adjustment for multiple comparisons
☐	☒	A full description of the statistical parameters including central tendency (e.g. means) or other basic estimates (e.g. regression coefficient) AND variation (e.g. standard deviation) or associated estimates of uncertainty (e.g. confidence intervals)
☒	☐	For null hypothesis testing, the test statistic (e.g. *F*, *t*, *r*) with confidence intervals, effect sizes, degrees of freedom and *P* value noted<br>*Give P values as exact values whenever suitable.*
☒	☐	For Bayesian analysis, information on the choice of priors and Markov chain Monte Carlo settings
☒	☐	For hierarchical and complex designs, identification of the appropriate level for tests and full reporting of outcomes
☒	☐	Estimates of effect sizes (e.g. Cohen's *d*, Pearson's *r*), indicating how they were calculated

Our web collection on statistics for biologists contains articles on many of the points above.

Software and code

Policy information about availability of computer code

Data collection	RNA Sequencing reads were obtained using the Illumina Novaseq 6000. GraphPad PRISM 9.0 was used to generate Volcano plots for RNA seq data and associated statistic analysis. GraphPad PRISM 9.0 was used to generate graphs for Immunofluorescence staining assays,Western blot histograms and associated statistic analysis.
Data analysis	Differential analysis was performed using DESeq2. Gene Ontology was performed using Metascape.

For manuscripts utilizing custom algorithms or software that are central to the research but not yet described in published literature, software must be made available to editors and reviewers. We strongly encourage code deposition in a community repository (e.g. GitHub). See the Nature Portfolio guidelines for submitting code & software for further information.

Data

Policy information about availability of data

All manuscripts must include a data availability statement. This statement should provide the following information, where applicable:

- Accession codes, unique identifiers, or web links for publicly available datasets
- A description of any restrictions on data availability
- For clinical datasets or third party data, please ensure that the statement adheres to our policy

All NGS data generated for the project GSE303280 have been deposited at the Gene Expression Omnibus (GEO) under accession number: GSM9122346,

GSM9122347, GSM9122348, GSM9122349, GSM9122350.
Source data are provided with this paper.

Research involving human participants, their data, or biological material

Policy information about studies with human participants or human data. See also policy information about sex, gender (identity/presentation), and sexual orientation and race, ethnicity and racism.

Reporting on sex and gender	No Human Participants
Reporting on race, ethnicity, or other socially relevant groupings	No Human Participants
Population characteristics	*Describe the covariate-relevant population characteristics of the human research participants (e.g. age, genotypic information, past and current diagnosis and treatment categories). If you filled out the behavioural & social sciences study design questions and have nothing to add here, write "See above."*
Recruitment	*Describe how participants were recruited. Outline any potential self-selection bias or other biases that may be present and how these are likely to impact results.*
Ethics oversight	*Identify the organization(s) that approved the study protocol.*

Note that full information on the approval of the study protocol must also be provided in the manuscript.

Field-specific reporting

Please select the one below that is the best fit for your research. If you are not sure, read the appropriate sections before making your selection.

☒ Life sciences ☐ Behavioural & social sciences ☐ Ecological, evolutionary & environmental sciences

For a reference copy of the document with all sections, see nature.com/documents/nr-reporting-summary-flat.pdf

Life sciences study design

All studies must disclose on these points even when the disclosure is negative.

Sample size	For all in vitro experiments, we have a minimum of n=3 biological replicates. For RNA-seq, we have a minimum of n=2 biological replicates.
Data exclusions	No data were excluded in this study.
Replication	All the experiments presented in this work were obtained from a minimum of 3 independent biological replicates.
Randomization	For the in vitro experiments, randomization was not performed as the control and the experimental groups were predefined.
Blinding	Data collection and analysis were performed blind whenever possible.

Reporting for specific materials, systems and methods

We require information from authors about some types of materials, experimental systems and methods used in many studies. Here, indicate whether each material, system or method listed is relevant to your study. If you are not sure if a list item applies to your research, read the appropriate section before selecting a response.

Materials & experimental systems

n/a	Involved in the study	
☐	☒	Antibodies
☐	☒	Eukaryotic cell lines
☒	☐	Palaeontology and archaeology
☒	☐	Animals and other organisms
☒	☐	Clinical data
☒	☐	Dual use research of concern
☒	☐	Plants

Methods

n/a	Involved in the study	
☒	☐	ChIP-seq
☐	☒	Flow cytometry
☒	☐	MRI-based neuroimaging

Antibodies

Antibodies used	Rabbit monoclonal, Anti-Phospho-Histone H2A.X (Ser139), Cell Signaling Technology, Cat# 9718T, RRID: AB_2118009<br>Rabbit monoclonal; Anti-pCHK1 (ser317), Cell signaling Technology, Cat# 12302S, RRID: AB_2783865<br>Rabbit monoclonal, Anti-pRPA32 (ser4, ser8), Bethyl Lab A300-245A-M, RRID: AB_210547<br>Rabbit monoclonal, Anti-H3, Cell Signaling Technology, Cat# 12648, RRID: AB_2797978<br>Rabbit monoclonal, Anti-H3K27ac, Cell Signaling Technology Cat# 8173S, RRID: AB_10949503<br>Rabbit monoclonal, Anti-p300, Cell Signaling Technology, Cat# 70088, RRID: AB_2799773<br>Rabbit monoclonal, Anti-CBP, Cell Signaling Technology, Cat# 7389, RRID: AB_2616020<br>Rabbit monoclonal, Anti-BRCA1, EMD Millipore, Cat# 07-434, RRID: AB_2275035<br>Rabbit monoclonal, Anti-rad51, Abcam, Cat#ab63801, RRID: AB_1142428<br>Rabbit monoclonal, Anti-phospho-ATR (Cell Signaling, Cat#30632, RRID: AB_2798992<br>Rabbit monoclonal, Anti-p-53BP1, Cell Signaling Technology, Cat#2675, RRID: AB_490917<br>Rabbit polyclonal, Anti-p-53BP1, Cell Signaling Technology, Cat# NB100-304, RRID: RRID: AB_350221<br>Rabbit polyclonal, Anti-53BP1, Novus Biologicals, Cat# NB100-304, RRID: AB_3155974<br>Rabbit polyclonal, Anti-FANCD2, Novus Biologicals, Cat#NB100-182, RRID: AB_3149941<br>Rabbit polyclonal, Anti-RPA, Bethyl, Cat# A300-244A, RRID: AB_185548<br>Rabbit polyclonal, Anti-Human RAD51, CosmobioUSA, Cat# BAM-70-002-EX, RRID: AB_10709951<br>Rabbit polyclonal, Anti-p300, Abcam, Cat# ab10485, RRID: AB_297224<br>Mouse monoclonal, Anti-CHK1, Cell Signaling Technology Cat# 2360, RRID: AB_2080320<br>Mouse monoclonal, Anti-phospho-H2A.X (Ser139), EMD Millipore, Cat#05-636, Clone JBW301, RRID: AB_309864<br>Mouse monoclonal, Anti-BRCA2 (Ab-1), Sigma Aldrich, Cat# OP95, Clone 2B, RRID: AB_2067762<br>Mouse monoclonal, Anti-POLD3, Abnova, Cat# H00010714-M01, Clone 3E2, RRID: AB_606803<br>Mouse monoclonal, Anti-Vinculin, Sigma Aldrich, Cat# V9131, Clone hVIN-1, RRID: AB_477629<br>Mouse monoclonal, Anti-Cyclin-A, SantaCruz, Cat# sc-271682, Clone B-8, RRID: AB_10709300<br><br>Anti-rabbit IgG Fab2 Alexa Fluor ® 488, Molecular probes, Cat#4412S, RRID: AB_1904025<br>Anti-rabbit IgG Fab2 Alexa Fluor ®594, Molecular probes, Cat# 8889S, RRID: AB_2716249<br>Anti-mouse IgG Fab2 Alexa Fluor ® 488, Molecular probes, Cat# 4408S, RRID: AB_10694704<br>Anti-mouse IgG Fab2 Alexa Fluor ® 594, Molecular probes, Cat# 8890S, RRID: AB_2714182<br>Alexa Fluor 568 Goat Anti-Mouse IgG (H+L), Invitrogen Molecular Probes, Cat# A-11031, RRID: AB_144696<br>Alexa Fluor 488 Goat Anti-Rat IgG (H+L), Invitrogen Molecular Probes, Cat# A-11006, RRID: AB_141373<br>Mouse monoclonal purified Anti-BrdU, BD Biosciences, Cat# 347580, RRID: AB_400326<br>Rat monoclonal Anti-BrdU, Abcam, Cat# ab6326, RRID: AB_305426
Validation	All antibodies used in this study are commercially available and have been validated either by the manufacturer or by ourselves using appropriate negative control lysates.

Eukaryotic cell lines

Policy information about cell lines and Sex and Gender in Research

Cell line source(s)	All the NA-ATLL, J-ATLL and the Jurkat cell lines were provided by co-author Dr. Hilda Ye. The B-lymphoblastoid cell line (GM03798) was obtained from Coriell Cell Repository.
Authentication	STR analysis was performed for all cell lines that were obtained from sources other than ATCC.
Mycoplasma contamination	All cells were routinely tested for mycoplasma and all cells used in this study were free of mycoplasma contamination.
Commonly misidentified lines (See ICLAC register)	No commonly misidentified cell lines were used in this study.

Plants

Seed stocks	No plants were used in this study
Novel plant genotypes	No plants were used in this study
Authentication	No plants were used in this study

Flow Cytometry

Plots

Confirm that:

☒ The axis labels state the marker and fluorochrome used (e.g. CD4-FITC).

☒ The axis scales are clearly visible. Include numbers along axes only for bottom left plot of group (a 'group' is an analysis of identical markers).

☒ All plots are contour plots with outliers or pseudocolor plots.

☒ A numerical value for number of cells or percentage (with statistics) is provided.

Methodology

Sample preparation	Sample preparation was done using Click-iT™ Plus EdU Cell Proliferation Kit for Imaging, Alexa Fluor™ 488 dye
Instrument	Attune 3L NxT Cytometer (IMFC-4.17)
Software	Analysis and gating was done using FlowJo 10.10.0
Cell population abundance	Cell population abundance was quantified using Beckman Coulter Vi-Cell XR Cell Viability Analyzer and validated using manual hemocytometer.
Gating strategy	Gating strategy involved initial selection of the population of interest based on FSC vs SSC; further subsequently single cells were gated using FSC-A vs FSC-H to exclude doublets. Lastly population of interest was gated using EDU vs PI population for cell cycle kinetics and Annexin V + population to denote apoptotic population.

☒ Tick this box to confirm that a figure exemplifying the gating strategy is provided in the Supplementary Information.
